# Determination of Rhythmicity and Gestational Stage-Related Distribution of Blood Plasma Melatonin Concentrations in Donkey Mares

**DOI:** 10.3390/vetsci11070310

**Published:** 2024-07-11

**Authors:** Levente Harmat, János Nagy, Bence Somoskői, Alán Alpár, Sándor György Fekete, András Gáspárdy

**Affiliations:** 1 Experimental Farm, University of Veterinary Medicine Budapest, Dóra-major, 2225 Üllő, Hungary; harmat.levente@univet.hu; 2 Game Management Landscape Centre, Hungarian University of Agriculture and Life Sciences, Kaposvár Campus, Malom utca 3, 7475 Bőszénfa, Hungary; nagy.janos@uni-mate.hu; 3 Department of Obstetrics and Food Animal Medicine Clinic, University of Veterinary Medicine Budapest, István utca 2, 1078 Budapest, Hungary; somoskoi.bence@univet.hu; 4 Department of Anatomy, Histology and Embryology, Semmelweis University, Tűzoltó utca 58, 1094 Budapest, Hungary; alpar.alan@semmelweis.hu; 5 SE NAP Research Group of Experimental Neuroanatomy and Developmental Biology, Semmelweis University, Tűzoltó utca 58, 1085 Budapest, Hungary; 6 Institute for Animal Breeding, Nutrition and Laboratory Animal Science, University of Veterinary Medicine Budapest, István utca 2, 1078 Budapest, Hungary; fekete.sandor@univet.hu

**Keywords:** circannual and circadian rhythms, gestation age, domestic donkey, blood melatonin

## Abstract

**Simple Summary:**

The domestic donkey (*Equus asinus*), like the horse (*Equus caballus*), is a short-day, seasonally polyestrous animal with an endogenous circannual reproduction rhythm. The main seasonal changes (winter–summer) significantly influence the development of the internal rhythm, which is directly controlled by melatonin. However, very little is known about the development of melatonin concentrations in donkey blood plasma. This study confirmed the circannual and circadian rhythms of the domestic donkey. It can be established that the lowest blood plasma melatonin concentration is typical in the Northern Hemisphere during the summer solstice. The significantly strongest melatonin production can be observed during the night of the winter solstice. The blood plasma melatonin concentration does not change as pregnancy progresses.

**Abstract:**

The aim of this study is to obtain a more complete picture of blood plasma melatonin concentrations in the donkey mares. To this purpose, sampling and statistical processing were carried out in such a way that allowed the researchers to establish the annual and daily rhythms. Based on human observations, according to the hypothesis of the authors, the blood plasma melatonin concentration of pregnant individuals rises during the late gestational period, before parturition. To confirm this, the melatonin concentrations of pregnant and non-pregnant jennies were monitored and compared. In regard to the circannual rhythm, the significantly lowest midnight melatonin value (27.67 pg mL^–^^1^) was typical for the summer solstice. Under consideration of circadian changes, a significantly strongest melatonin production (45.16 pg mL^–^^1^) was observed on the night of the winter solstice (*p* < 0.001). Considering gestational age, the blood plasma melatonin concentration (around 38 pg mL^–^^1^) does not change as gestation progresses (*p* = 0.136). The results obtained in this studied population of the domestic ass usefully expand the little knowledge previously gathered about the development of the blood plasma melatonin concentrations of this species.

## 1. Introduction

Melatonin is the regulator of the body’s daily and annual physiological cycles, and its role is especially important in reproductive processes, both in animals and humans. At the same time, melatonin production, influenced by the duration of illumination, determines not only seasonal and daily rhythms but also changes in the reproductive system. Furthermore, it has antioxidant and immunomodulating properties and is also involved in some metabolic processes [1].

The light stimulus enters from the melanopsin-producing ganglion cells of the retina to the suprachiasmatic nucleus of the hypothalamus via the retinohypothalamic tract. Then, it reaches the pineal gland (epiphysis) via the sympathetic noradrenergic pathway, where melatonin is synthesized [2]. Light inhibits the production and secretion of the hormone, so the highest concentrations of melatonin can be measured during darkness. Melatonin is not only produced in the pineal gland, but extrapineal melatonin has also been detected in various organs: the gastrointestinal tract [3], liver [4], kidney, heart, thymus [5], adrenal gland [6], brain [7], gonads, placenta, uterus [8], platelets, white blood cells, and other cells of the immune system [9]. The synthesis of melatonin in the mitochondria of eukaryotic cells represents a unique protection against oxidative damage and plays an important role in maintaining the physiological functioning of cells [10].

The melatonin produced in the pineal gland modulates the functioning of the pituitary gland, including the production of oxytocin, vasopressin, and prolactin. In addition, melatonin affects sexual development and reproductive processes and activates the hypothalamus–pituitary–ovary axis (HPO) system [11]. The presence of melatonin is sensed by its receptors on the surface of the cells of both central nervous and peripheral tissues. Of the two types of melatonin receptors (MT1 and MT2) in mammals, MT1 is significantly more important in melatonin-controlled reproductive processes [12].

In horses and donkeys, short days inhibit the reproductive cycle through the HPO axis. During the non-estrous period, the hypothalamus’ gonadotropin-releasing hormone (GnRH) [13] and the pituitary gland’s luteinizing hormone (LH) secretions [14] are low. The production of melatonin is an important indicator of the maintenance of anestrus. Removal of the pineal gland [15] or surgical transection of its sympathetic innervation [16], by interfering with melatonin production, delays the onset of the breeding season [17].

The domestic donkey (*Equus asinus*), similar to the horse (*Equus caballus*), is a seasonally polyestrous animal and has an endogenous circannual reproductive rhythm, and the main seasonal changes (winter–summer) have a significant influence on the development of its internal rhythm, for which melatonin is the “calendar information” organization. The main regulator is the increasing daylight in spring, which reactivates the HPO axis as a seasonal change and results in a reduced melatonin concentration [18,19]. At the same time, the natural physiological breeding season of the donkey lasts from March to May in the Northern Hemisphere, and its formation is influenced by the lengthening of the days, the elevation of the temperature, and the energy content of the feed. Year-round active cycles can be observed at the equator. Both the estrus and the pregnancy periods are longer in the jennies (by 1–2 and 37–38 days, respectively) than in horse mares. Ovulation is spontaneous, it occurs on the 2nd–4th day estrus. The first estrus can be observed 9–11 days after foaling [20]. The length of pregnancy is one year and sometimes a little more when jennies become pregnant at the early period [21]. It is recommended to take advantage of first estrus for conception, especially if we want to keep a short interval between two foalings [22]. Although information on mule pregnancy is sporadic, in practice, both interspecies hybrids (mules and hinnies) are infertile due to different parental chromosome numbers. Nevertheless, the mule’s melatonin production also shows seasonality; in autumn, higher melatonin concentrations appear in the blood than in spring [23].

The increase in the number of long days and light hours affects not only the triggering of ovulation and the start of the cycle but also the shedding of the winter coat and the time of foaling. The fetus developing inside the mother’s body is initially under the hormonal control of the mother animal and is part of its internal balance. This is how it is affected by the mother’s body temperature and the maternal hormones that are able to reach the fetus through the placenta [24]. It is known that some representatives of the prolactin and growth hormone (GH) families pass into the mammalian fetus and enter circulation there. The gradually increasing daily light in spring causes a decrease in melatonin production and an increase in the concentration of circulating prolactin in horses [24].

In a study by Altinsaat et al. (2009), the melatonin concentrations of horse mares and stallions in June (15 h light–9 h dark) were lower (23.52 and 17.22 pg mL^–^^1^) than in December (9 h light–15 h dark) (42.41 and 37.68 pg mL^–^^1^) [25]. The night serum melatonin concentration in horse mares is between 10 and 20 pg mL^–^^1^ based on the experience of several authors [26,27,28], with the exception of a few outliers (around 50 pg mL^–^^1^). In a study conducted by Guillaume et al. (2006), in jennies, the nocturnal melatonin concentration in autumn was on average 90 pg mL^–^^1^ [29]. The same authors reported even higher levels (average of 169 pg mL^–^^1^) in mule mares, confirming the previous evaluations of others [23]. So, donkeys have a higher average plasma melatonin concentration than horses. At the same time, the melatonin concentrations in the seminal plasma of donkey and horse stallions obtained during the breeding season (March to June) were similar but with low values (2.82 and 2.48 pg mL^–^^1^, respectively) [30]. In Brazil, Messias et al. (2022) measured the concentrations of melatonin in donkey milk collected during morning milking as 4–5 pg mL^–^^1^ [31].

The circadian rhythm of melatonin secretion plays an important role in maintaining the fetus, having an anti-inflammatory effect. As shown in humans, melatonin participates in the processes of implantation, the development of the placenta, and the maintenance of the neuroimmunoendocrine processes of the placenta, the purpose of which is the development of the vital functioning systems of the fetus. By regulating apoptosis, melatonin maintains the balance of cyto- and syncytiotrophoblast cells, thereby promoting homeostasis of the placenta [32]. Melatonin produced in the placenta is secreted into the amniotic fluid, where it exerts antioxidant, anti-inflammatory, and analgesic effects [33,34]. In humans, blood melatonin levels increase during pregnancy, especially after 24 weeks, and reach their highest concentration before labor [35,36]. The circadian rhythm of gene expression has been established directly in the uterus of pregnant and non-pregnant rodents, which highlights the fact that melatonin triggers labor and parturition as a circadian signal [37]. Labor begins in the late evening or early morning when melatonin secretion increases [38].

The donkey is regarded as an autochthonous animal species in Hungary. In 2003, its breeding association was founded, and its stud book contains about 4000 individuals with limited ancestry information. The utilization of donkeys in Hungary previously was principally linked to the activities of the herding and keeping of sheep [39]. The main reasons for using donkeys were for their high social ability, the protection of livestock, use as pack animals, and their ability to thrive on poor-quality pasture. This domestic donkey population is heterogenous in appearance, and the breed-specific characteristics are less typical than in other European donkey breeds [40]. There is a difference in body capacity according to color variants; the grey animals are of significant less body capacity than the brown and black ones. The height at the withers is about 117 cm on average; however, it varies in a large range. In order to unify the Hungarian Fallow Donkey breed, a reconstruction program began in 2021 [41].

To the best of our knowledge, studies conducted so far on the natural blood melatonin concentrations in donkeys are limited to this literature.

The aim of this study is to obtain a more complete picture of blood plasma melatonin concentrations in the donkey species. The authors’ assumption is that this species also has the cyclicity typical of horses. To this end, sampling and statistical processing were carried out in such a way that the annual and daily rhythms could be established. According to the authors’ second hypothesis, the blood plasma melatonin concentration of pregnant donkey mares increases in the late pregnancy period, as indicated by human observations. To prove this, the melatonin concentrations of pregnant and non-pregnant mares were monitored and compared. 

## 2. Materials and Methods

### 2.1. Study Design

The project was approved under animal experiment license number PE/EA/01444-6/2022. The study was conducted in the nucleus stud farm of Hungarian Fallow Donkeys located in Bőszénfa (Game Management Landscape Centre, Kaposvár Campus, Hungarian University of Agriculture and Life Sciences). The stud farm is located at a latitude of 46.23 and a longitude of 17.85. The average temperature in December and in June is 2 and 20 degrees Celsius, respectively. The times of the sunrise and sunset at the winter solstice are 07:29 and 16:05, and at the summer solstice, they are 04:56 and 20:45. All jennies were kept in free-range conditions almost year-round and spent the day and night in the pasture. During wintertime, their feed was supplemented with hay. They always had access to water. The studs’ health was impeccable. The body condition of the animals was repeatedly determined using visual inspection and manual palpation according to the guidelines of The Donkey Sanctuary [42]. Scores varied between 3 (ideal, two-thirds of observations) and 4 (fat). Thus, the animals were well or slightly over-nourished. A thick neck makes finding the jugular vein and blood sampling difficult. The basic criterion for keeping them in breeding is the existence of natural resistance, so no treatment against parasites is carried out. Individuals showing a deteriorating condition and infertility are culled. The joining period is limited to April and May. The re-breeding occurs through natural service in harems.

The day before the sampling, all animals were herded into their free-stall barn and kept there until the sampling was completed. For the examination, 15 mares were selected according to their pregnancy status; otherwise, they were selected randomly at the beginning, and the same ones remained for continued monitoring. By mid-July, the pregnant jennies were growing approximately two-month-old fetuses. The pregnancy status of the individuals was then determined with a transrectal ultrasound scanner (Easy-Scan:Go, IMV Imaging Ltd., Bellshill, UK). Sampling took place three times at predetermined times: 14 July 2022, 12–13 November 2022, and 16–17 April 2023. All three sampling occasions covered a 24 h period; that is, a new individual was sampled every hour and a half. The individuals participating in the study lived together with the stud during the entire study period. The median age of the individuals in the study was 9 years old (with years of birth between 2001 and 2017).

### 2.2. Sampling and Determination of Melatonin

In the stable, the light intensity was always below 10 Lux at night and 100 Lux during the day, which was determined with a handy light meter (Testo 540 Part no. 0560 0540/0520 0010, Testo Ltd., Hampshire, UK). The night sampling was performed with use of a red light at a low illuminance near the animal. For daytime sampling, the animals remained in the barn, so that melatonin production remained at a detectable level.

About 8 mL of blood was taken from the jugular vein into vacutainers containing EDTA (Premium Vacuette^®^ K3E K3EDTA Blood Collection Tube, Ref: 454086, Greiner Bio-One GmbH, Kremsmünster, Austria) when sampling. Then samples were kept below 5 °C. Within a day, samples were centrifuged using a universal centrifuge (Z 326 K, HERMLE Labortechnik GmbH, Wehingen, Germany) at 2000× *g* for 10 min. After harvesting plasma into Eppendorf tubes, it was immediately frozen and stored at −18 °C. For the determination of melatonin concentrations (pg mL^–^^1^), a Tecan RE29301 RIA kit (RE29301, IBL International GmbH, Hamburg, Germany) containing an iodine-125 radioisotope-labeled anti-melatonin antibody (indirect radioimmunoassay) was used according to the manufacturer’s instructions. Samples (200 µL) were measured in duplicates and their average calculated. Measurements were made by using a gamma counter (Perkin Elmer Wallac Wizard 1470, Wallac Oy, Turku, Finland).

### 2.3. Statistical Processing

Melatonin test results were pooled, and then a Kolmogorov–Smirnov one-sample test for normality was performed. This test confirmed the normal distribution of the melatonin data (d = 0.1257, *p* > 0.20).

As a background data set for processing of the melatonin results, the following variables were recorded and calculated:

- Identification number: the equine national life number, which was established by the use of a microchip reader;
- Pedigree: the origin of the mares was known to the extent of two ancestral rows;
- Sampling date: the day when the blood was collected;
- Sampling time: the hour and minute of blood collection;
- Days around winter solstice: the number of days between 21 December 2022, and the date of sampling (in a range between −160 and 128);
- Minutes around midnight: the period (in minutes) between midnight and exact taking of blood sample (in a range between −715 and 710);
- Pregnancy status: it was determined with ultrasound examination in 2022: pregnant (n = 8) or non-pregnant mares (n = 7);
- Foaling date: the day when parturition occurred;
- Days to foaling: It was the inverse of the gestational age, with the time between the date of foaling and the date of sampling expressed in days. This approach can be considered favorable due to the variable length of pregnancy. The non-pregnant mares were given a simulated foaling of May 15, representing the average date of foaling for this stud.

The statistical processing of the obtained melatonin concentrations was realized in two steps. First, they were corrected by eliminating the factors affecting them. With the help of an individual animal model (Version 6.5f, Pedigree Viewer, University of New England, Sidney, Australia), the effect of the serial number of the sampling and the pregnancy status (as fixed effects) and the distance from the winter solstice, midnight, and foaling (as covariates) were filtered out [43]. This is how the corrected values of the melatonin concentrations were created, which were graphically illustrated according to circannual rhythm, circadian rhythm, and throughout gestation. During the correction, the effect on which the course of melatonin concentration was presented was removed from the model.

In the second step, adjusted values were estimated for different specific times (transitions between seasons, parts of the days, and stages of lactation) on the corrected values by use of linear regression. So, adjustments were carried out for summer and winter solstices and autumnal and vernal equinoxes. In regard to the day, adjusted values were computed for midday, 18:00 in the afternoon, midnight, and 6:00 in the morning. Regarding pregnancy, adjusted values were prepared for 300, 150, and 5 days before foaling. Adjusted values were also shown graphically, and additionally, distance-weighted least squares curves were fitted depending on the circannual rhythm, circadian rhythm, and gestation. A statistical test (one-way ANOVA with a Tukey honest significant difference test) was performed to compare them at midnight. As result, the mean and SEM (standard error of the mean) were displayed. For the calculation of the regression, statistical differences, and graphical presentation, Statistica software (version 14., TIBCO Software Inc., Palo Alto, CA, USA) was applied [44].

## 3. Results

### 3.1. Circannual Rhythm

**Figure 1:**
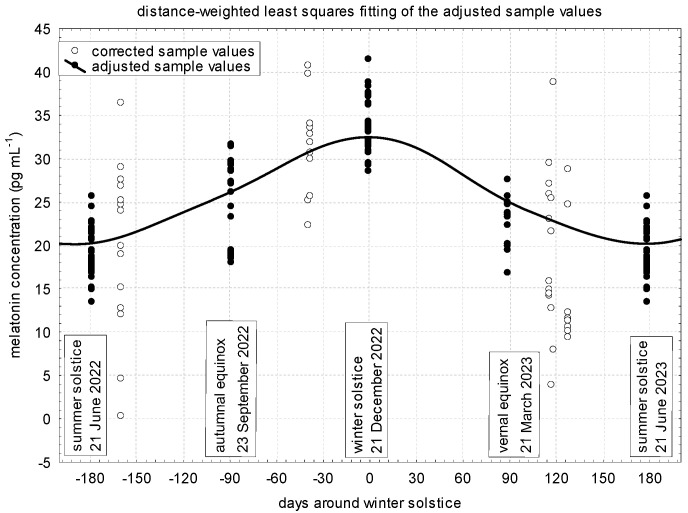
Yearly distribution of plasma melatonin concentrations in the investigated donkey population (pregnant and non-pregnant animals combined).

The corrected values for each sampling, the values adjusted for transitions between seasons, and the estimated annual distribution of melatonin concentrations are presented in Figure 1. In the mare study population, the highest melatonin concentration was found on the winter solstice (about 32–33 pg mL^–^^1^) and the lowest one on the summer solstice (about 20 pg mL^–^^1^), while during the autumn and spring equinoxes, the concentrations of melatonin were roughly the same (27 and 25 pg mL^–^^1^, respectively), time proportionally, between the values of the two solstices.

Melatonin concentrations adjusted for the transitions between seasons and their statistical tests are presented in Table 1. These values reflect the previously determined values, with the difference that they are higher because these values are also adjusted for midnight. The significantly lowest value (27.67 pg mL^–^^1^) was typical during the summer solstice at midnight. During the autumn and spring equinoxes, similar concentrations (33.35 and 31.23 pg mL^–^^1^, respectively), but significantly different from the values at the solstices, were established. On the night of the winter solstice, the highest melatonin concentration (42.18 pg mL^–^^1^) was formed in the blood plasma.

### 3.2. Circadian Rhythm

**Figure 2:**
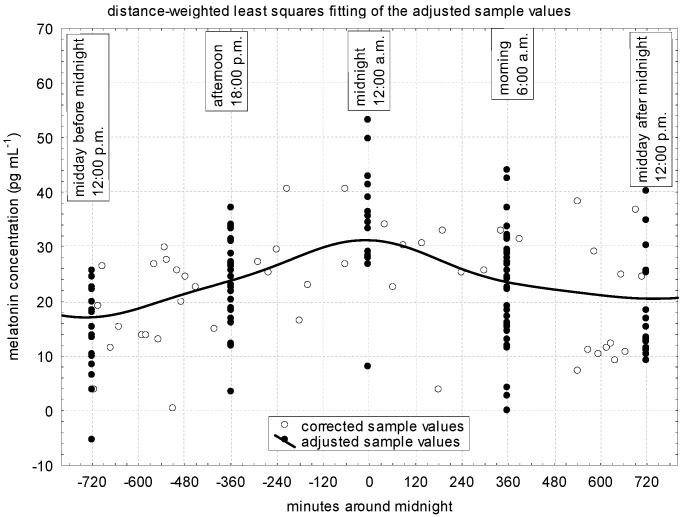
Daily distribution of plasma melatonin concentrations in the investigated donkey population (pregnant and non-pregnant animals combined).

In Figure 2, the corrected values for each sampling, the values adjusted for times of the day, and the estimated circadian distribution of the melatonin concentrations are presented. In the mares investigated, the highest melatonin concentration was observed at midnight (about 32 pg mL^–^^1^) and the lowest ones at noon before and after midnight (±720 min from midnight; about 17 and 20 pg mL^–^^1^, respectively). At the same time, 6–6 h (equivalent to ±360 min) before and after midnight, the concentrations of melatonin were similar (24 and 23 pg mL^–^^1^, respectively).

Melatonin concentrations, adjusted for the times of the day and the winter solstice with statistical tests, are presented in Table 2. These values correspond to the previously presented values (see Figure 2). The values at noon (23.85 and 28.81 pg mL^–^^1^, respectively) were significantly the lowest. The afternoon and morning values were in the medium range (33.17 and 32.63 pg mL^–^^1^, respectively). In accordance with the previous section (see Table 1), the strongest melatonin production (45.16 pg mL^–^^1^) was observed on the night of the winter solstice. Now, we emphasize that the values can be interpreted by themselves, per processing.

### 3.3. Gestational Age

**Figure 3:**
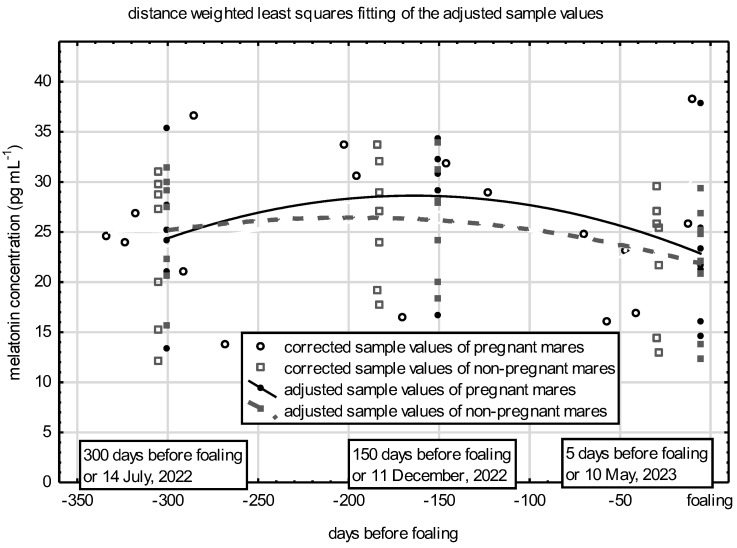
Distribution of plasma melatonin concentrations during gestation in pregnant mares and in non-pregnant controls.

The corrected values for each sampling, the values adjusted for specific days of gestation, and the estimated distribution of melatonin concentrations during the last 300 days before foaling are presented in Figure 3. The melatonin concentration of pregnant mares during this period of pregnancy can be considered constant at around 25 pg mL^–^^1^. The values of the non-pregnant animals also stagnated at the same level during the control period.

There were no confirmed differences between the melatonin concentration values calculated at midnight during the study period, either according to gestational age or pregnancy status (Table 3). These values were around 38 pg mL^–^^1^ and did not change as pregnancy progressed (*p* = 0.136). The apparent change can be regarded as a residual error despite correction for the season.

## 4. Discussion

The peak concentration of blood plasma melatonin in the jennies was 45 pg mL^–^^1^ under the study circumstances (at midnight on the winter solstice, Northern Hemisphere). This is roughly half of what was previously found [29].

We determined the changes in blood plasma melatonin concentrations during a whole year. Regarding the autumn period, Guillaume et al. (2006) also detected an increase in nocturnal melatonin concentrations during two consecutive weeks [29]. According to Cozzi et al. (1991), the mule’s melatonin production also shows cyclicity: in autumn, higher melatonin concentrations appear in the blood than in spring [23]. The same can be established in the present study, although the difference was not significant. However, the difference may have a physiological background since an intense decrease in melatonin concentration at the beginning of the year is a prerequisite for the start of the early reproductive period and estrus in donkeys.

The daily rhythm of blood plasma melatonin concentrations was also confirmed in this study. However, it should be emphasized that the daytime brightness never exceeded 100 Lux during the examination. Otherwise, the production of melatonin would have decreased below the undetectable concentration (<0.5 pg mL^–^^1^), as was presented in the case of horses [45]. A study conducted by Cozzi et al. (1991) on mules showed the circadian rhythm of melatonin production too [23]. The rise in melatonin production started at 18 p.m. in fall and 22 p.m. in spring. Interestingly, the drop in melatonin production occurred from around 7 a.m. to 8 a.m., at the same time in both fall and spring. This study of mules showed that melatonin production follows the natural pattern of the daily light–dark cycle. In the horse species, several studies [26,46] also showed a significant 24 h rhythm of plasma melatonin levels under natural conditions, i.e., a continuous light period of several hours is followed by a continuous dark period of several hours, and the sum of these constitutes the whole day. Taking samples every two hours from horses kept in darkness for 24 h, Murphy et al. (2011) found no change in serum melatonin concentrations over time [47]. Based on those data, they concluded that melatonin is not the only thing that maintains circadian processes in horses.

The last sample collection took place within two months before foaling. Corrections were made for a more reliable prenatal time (5 days before parturition) but not for more uncertain parturition day. Knowledge of melatonin concentrations immediately before parturition and at the time of parturition requires further investigations. There is information about the nocturnal melatonin concentration in horse mares at parturition. Having the average foaling day at the beginning of March, this amounted to 36.35 pg mL^–^^1^ [48].

## 5. Conclusions

The present circannual and circadian determinations of melatonin concentrations were the first to be performed on mares of the donkey species and led to important new results. It was found that the peak concentration of melatonin was 45 pg mL^–1^. Daily and annual rhythms were formed in the jennies since values tended to be lower than this in the other seasons and times of the day. These findings show the great similarity of the donkey to the horse in the geographical area of Central Europe.

Since the donkey population was housed indoors during the experiment and the daylight remained below 100 Lux, melatonin values could also be measured during the day. However, as a result, the circannual and circadian rhythms appeared in a narrower range. In greater daylight, the values would probably have been scattered in a wider range from below.

It has been confirmed that the concentration of melatonin does not change during the gestation period, unlike human findings. Detailed determination of melatonin concentrations around foaling still awaits as valuable research.

## Figures and Tables

**Table 1:** Melatonin concentrations adjusted for the transitions between seasons and midnight in the investigated jennies (pregnant and non-pregnant animals combined).

Event *p* < 0.001	Mean Melatonin Concentration, pg mL^–1^	SEM *
Summer solstice, 22 June 2022, 12 a.m.	27.67 ^a^	0.459
Autumnal equinox, 24 September 2022, 12 a.m.	33.35 ^b^	1.071
Winter solstice, 22 December 2022, 12 a.m.	42.18 ^c^	0.523
Vernal equinox, 22 March 2023, 12 a.m.	31.23 ^b^	0.683

* Standard error of mean. ^a–c^ different superscript letters differ significantly (Tukey honest significant difference test, *p* < 0.05).

**Table 2:** Daily melatonin concentrations adjusted for the times of the day and the winter solstice in the investigated jennies (pregnant and non-pregnant animals combined).

Parts of the Day *p* < 0.001	Mean Melatonin Concentration, pg mL^–1^	SEM *
Midday before midnight, 12:00 p.m., 21 December 2022	23.85 ^a^	1.981
Afternoon, 18:00 p.m., 21 December 2022	33.17 ^b^	1.607
Midnight, 12:00 a.m., 22 December 2022	45.16 ^c^	2.460
Morning, 06:00 a.m., 22 December 2022	32.63 ^b^	1.652
Midday after midnight, 12:00 p.m., 22 December 2022	28.81 ^ab^	2.371

* Standard error of mean. ^a–c^ different superscript letters differ significantly (Tukey honest significant difference test, *p* < 0.05).

**Table 3:** Melatonin concentrations adjusted for midnight according to specific days of gestation.

Days to Foaling *p* = 0.136	Mean Melatonin Concentration, pg mL^–1^	SEM *
300 days before foaling, *p* = 0.830	38.80	1.74
Pregnant	38.37	2.971
Non-pregnant	39.17	2.218
150 days before foaling, *p* = 0.523	41.17	1.757
Pregnant	42.58	3.107
Non-pregnant	40.16	2.169
5 days before foaling, *p* = 0.771	36.09	1.754
Pregnant	36.67	2.922
Non-pregnant	35.58	2.248

* Standard error of mean.

## Data Availability

The data that support the findings of this study are available from the corresponding author upon reasonable request.

## References

[1] Olcese J.M. (2020). Melatonin and Female Reproduction: An Expanding Universe. Front. Endocrinol..

[2] Schroeder M.M., Harrison K.R., Jaeckel E.R., Berger H.N., Zhao X., Flannery M.P., St Pierre E.C., Pateqi N., Jachimska A., Chervenak A.P. (2018). The roles of rods, cones, and melanopsin in photoresponses of M4 intrinsically photosensitive retinal ganglion cells (ipRGCs) and optokinetic visual behavior. Front. Cell. Neurosci..

[3] Bubenik G.A. (2002). Gastrointestinal melatonin: Localization, function, and clinical relevance. Dig. Dis. Sci..

[4] Sato K., Meng F., Francis H., Wu N., Chen L., Kennedy L., Zhou T., Franchitto A., Onori P., Gaudio E. (2020). Melatonin and circadian rhythms in liver diseases: Functional roles and potential therapies. J. Pineal Res..

[5] Sanchez-Hidalgo M., Alarcon de la Lastra C., Carrascosa-Salmoral M.P., Naranjo M.C., Gomez-Corvera A., Caballero B., Guerrero J.M. (2009). Age-related changes in melatonin synthesis in rat extrapineal tissues. Exp. Gerontol..

[6] Campino C., Valenzuela F.J., Torres-Farfan C., Reynolds H.E., Abarzua-Catalan L., Arteaga E., Trucco C., Guzmán S., Valenzuela G.J., Seron-Ferre M. (2011). Melatonin Exerts Direct Inhibitory Actions on ACTH Responses in the Human Adrenal Gland. Horm. Metab. Res..

[7] Jimenez-Jorge S., Guerrero J.M., Jimenez-Caliani A.J., Naranjo M.C., Lardone P.J., Carrillo-Vico A., Osuna C., Molinero P. (2007). Evidence for melatonin synthesis in the rat brain during development. J. Pineal Res..

[8] Reiter R.J., Tan D.-X., Manchester L.C., Paredes S.D., Mayo J.C., Sainz R.M. (2009). Melatonin and Reproduction Revisited. Biol. Reprod..

[9] Kvetnoy I.M. (1999). Extrapineal melatonin: Location and role within diffuse neuroendocrine system. Histochem. J..

[10] Bartha B., Harmat L., Somoskői B., Cseh S., Fekete S.G., Gáspárdy A. (2021). The role of melatonin in horse and donkey reproduction. Literature review. Hung. Vet. J..

[11] Talpur H.S., Chandio I.B., Brohi R.D., Worku T., Rehman Z., Bhattarai D., Ullah F., JiaJia L., Yang L. (2018). Research progress on the role of melatonin and its receptors in animal reproduction: A comprehensive review. Reprod. Domest. Anim..

[12] Gao Y., Zhao S., Zhang Y., Zhang Q. (2022). Melatonin Receptors: A Key Mediator in Animal Reproduction. Vet. Sci..

[13] Sharp D.C., Grubaugh W.R. (1987). Use of push-pull perfusion techniques in studies of gonadotropin-releasing hormone secretion in mares. J. Reprod. Fertil. Suppl..

[14] Hart P.J., Squires E.L., Imel K.J., Nett T.M. (1984). Seasonal variation in hypothalamic content of gonadotropin-releasing hormone (GnRH), pituitary receptors for GnRH, and pituitary content of luteinizing hormone and follicle-stimulating hormone in the mare. Biol. Reprod..

[15] Grubaugh W., Sharp D.C., Berglund L.A., McDowell K.J., Kilmer D.M., Peck L.S., Seamans K.W. (1982). Effects of pinealectomy in Pony mares. J. Reprod. Fertil. Suppl..

[16] Sharp D.C., Vernon M.W., Zavy M.T. (1979). Alteration of seasonal reproductive patterns in mares following superior cervical ganglionectomy. J. Reprod. Fertil. Suppl..

[17] Kilmer D.M., Sharp D.C., Berglund L.A., Grubaugh W., McDowell K.J., Peck L.S. (1982). Melatonin rhythms in Pony mares and foals. J. Reprod. Fertil. Suppl..

[18] Nagy P., Guillaume D., Daels P. (2000). Seasonality in mares. Anim. Reprod. Sci..

[19] Murphy B.A. (2019). Circadian and circannual regulation in the horse: Internal timing in an elite athlete. J. Equine Vet. Sci..

[20] Fielding D. (1988). Reproductive characteristics of the jenny donkey—*Equus asinus*: A review. Trop. Anim. Health Prod..

[21] Galisteo J., Perez-Marin C.C. (2010). Factors affecting gestation length and estrus cycle characteristics in Spanish donkey breeds reared in southern Spain. Theriogenology.

[22] Carluccio A., Gloria A., Robbe D., Veronesi M.C., De Amicis I., Cairoli F., Contri A. (2017). Reproductive characteristics of foal heat in female donkeys. Animal.

[23] Cozzi B., Morei G., Ravault J.P., Chesneau D., Reiter R.J. (1991). Circadian and seasonal rhythms of melatonin production in mules (*Equus asinus* × *Equus caballus*). J. Pineal Res..

[24] O’Brien C., Darcy-Dunne M.R., Murphy B.A. (2020). The effects of extended photoperiod and warmth on hair growth in ponies and horses at different times of year. PLoS ONE.

[25] Altinsaat Ç., Üner A.G., Sulu N., Ergün A. (2009). Seasonal variations in serum concentrations of melatonin, testosterone, and progesterone in Arabian horse. Ankara Üniv. Vet. Fak. Derg..

[26] Guerin M.V., Deed J.R., Kennaway D.J., Matthews C.D. (1995). Plasma melatonin in the horse: Measurements in natural photoperiod and in acutely extended darkness throughout the year. J. Pineal Res..

[27] Haritou S.J.A., Zylstra R., Ralli C., Turner S., Tortonese D.J. (2008). Seasonal changes in circadian peripheral plasma concentrations of melatonin, serotonin, dopamine and cortisol in aged horses with Cushing’s disease under natural photoperiod. J. Neuroendocrinol..

[28] Rapacz A., Lewczuk B., Prusik M., Raś A. (2010). Diurnal rhythm of plasma melatonin level in mares from spring equinox to summer solstice. Bull. Vet. Inst. Pulawy.

[29] Guillaume D., Zarazaga L., Malpaux B., Chemineau P. (2006). Variability of plasma melatonin level in pony mares (*Equus caballus*), comparison with the hybrid: Mules and with jennies (*Equus asinus*). Reprod. Nutr Dev..

[30] González-Arto M., Vicente-Carrillo A., Martínez-Pastor F., Fernández-Alegre E., Roca J., Miró J., Rigau T., Rodríguez-Gil J.E., Pérez-Pé R., Muiño-Blanco T. (2016). Melatonin receptors MT1 and MT2 are expressed in spermatozoa from several seasonal and nonseasonal breeder species. Theriogenology.

[31] Messias T.B.O.N., Sant’Ana A.M.S., Araújo E.O.M., Rangel A.H.N., Vasconcelos A.S.E., Salles H.O., Morgano M.A., Silva V.S.N., Pacheco M.T.B., Queiroga R.C.R.E. (2022). Milk from Nordestina donkey breed in Brazil: Nutritional potential and physicochemical characteristics in lactation. Int. Dairy J..

[32] Reiter R.J., Rosales-Corral S.A., Manchester L.C., Tan D.-X. (2013). Peripheral reproductive organ health and melatonin: Ready for prime time. Int. J. Mol. Sci..

[33] Tarocco A., Caroccia N., Morciano G., Wieckowski M.R., Ancora G., Garani G., Pinton P. (2019). Melatonin as a master regulator of cell death and inflammation: Molecular mechanisms and clinical implications for newborn care. Cell Death Dis..

[34] Wilhelmsen M., Amirian I., Reiter R.J., Rosenberg J., Gönegur I. (2011). Analgesic effects of melatonin: A review of current evidence from experimental and clinical studies. J. Pineal Res..

[35] Kivelä A. (1991). Serum melatonin during human pregnancy. Acta Endocrinol..

[36] Ejaz H., Figaro J.K., Woolner A.M.F., Thottakam B.M.V., Galley H.F. (2021). Maternal Serum Melatonin Increases During Pregnancy and Falls Immediately After Delivery Implicating the Placenta as a Major Source of Melatonin. Front. Endocrinol..

[37] Olcese J. (2012). Circadian aspects of mammalian parturition: A review. Mol. Cell. Endocrinol..

[38] Cagnacci A., Soldani R., Melis G.B., Volpe A. (1998). Diurnal rhythms of labor and delivery in women: Modulation by parity and seasons. Am. J. Obstet. Gynecol..

[39] Ernst J., Bodó I. (2004). The donkey. Living Heritage, Old Historical Hungarian Livestock.

[40] Lénárt Z., Ernst M., Gáspárdy A. (2017). Preliminary results on body conformation of Hungarian Fallow Donkey. Danub. Anim. Genet. Resour..

[41] Harmat L., Kuncicky A., Lénárt Z., Ernst M., Nagy J., Gáspárdy A. (2022). Conformation traits of Hungarian Fallow Donkey mares according to their basic colour. Danub. Anim. Genet. Resour..

[42] The Donkey Sanctuary. https://www.thedonkeysanctuary.org.uk.

[43] Pedigree Viewer. 2015. Version 6.5f. http://bkinghor.une.edu.au/pedigree.htm.

[44] TIBCO Software Inc. 2020. Data Science Workbench. Statistica Version 14. http://tibco.com.

[45] Murphy B.A., Elliott J.A., Sessions D.R., Vick M.M., Kennedy E.L., Fitzgerald B.P. (2007). Rapid phase adjustment of melatonin and core body temperature rhythms following a 6-h advance of the light/dark cycle in the horse. J. Circadian Rhythm..

[46] Piccione G., Giannetto C., Bertolucci C., Refinetti R. (2013). Daily rhythmicity of circulating melatonin is not endogenously generated in the horse. Biol. Rhythm Res..

[47] Murphy B.A., Martin A.-M., Furney P., Elliott J.A. (2011). Absence of a serum melatonin rhythm under acutely extended darkness in the horse. J. Circadian Rhythm..

[48] Gáspárdy A., Gallagher G., Bartha B., Cseh S., Fekete S.G., Somoskői B. (2023). Plasma melatonin concentration during the early post-partum period in Thoroughbred mares and their foals. Acta Vet. Hung..

